# ﻿Description of five larvae of the genus *Gnaptorina* Reitter, 1887 from Xizang, China (Coleoptera, Tenebrionidae, Blaptinae), with molecular species delimitation and diagnoses

**DOI:** 10.3897/zookeys.1209.124184

**Published:** 2024-08-14

**Authors:** Bao-Yue Ji, Tong-Yang Guo, Mei-Chang Gu, Guo-Dong Ren, Xiu-Min Li

**Affiliations:** 1 Key Laboratory of Zoological Systematics and Application of Hebei Province, College of Life Sciences, Institute of Life Science and Green Development, Hebei University, Baoding 071002, China Hebei University Baoding China

**Keywords:** Beetles, Gnaptorinina, immature stage, morphology, species identification

## Abstract

With 39 described species in three subgenera, the *Gnaptorina* is the second most species-rich genus in the subtribe Gnaptorinina (Tenebrionidae: Blaptinae). In this study, a phylogeny of *Gnaptorina* was reconstructed based on one nuclear (28S-D2) and three mitochondrial (COI, Cytb, and 16S) gene fragments; multiple molecular species delimitation approaches were also implemented to assess the taxonomic status of larval specimens based on COI gene fragment. Larvae of five known species of the subgenus Hesperoptorina are described and illustrated for the first time: *Gnaptorinanigera* Shi, Ren & Merkl, 2007, *Gnaptorinatishkovi* Medvedev, 1998, *Gnaptorinabrucei* Blair, 1923, *Gnaptorinahimalaya* Shi, Ren & Merkl, 2007, *Gnaptorinakangmar* Shi, Ren & Merkl, 2007. A key to larvae of four genera of the tribe Blaptini and a key to the known larvae of the genus *Gnaptorina* are provided. This study provides valuable morphological data for larval studies of the tribe Blaptini.

## ﻿Introduction

The Gnaptorinina Medvedev, 2001 is a species-rich subtribe of Blaptini Leach, 1815, consisting of 189 species in 12 genera: *Agnaptoria* Reitter, 1887 (36 species and subspecies), *Asidoblaps* Fairmaire, 1886 (56 species), *Blaptogonia* Medvedev, 1998 (five species), *Colasia* Koch, 1965 (seven species), *Gnaptorina* Reitter, 1887 (39 species and subspecies), *Itagonia* Reitter, 1887 (24 species and subspecies), *Montagona* Medvedev, 1998 (three species), *Nepalindia* Medvedev, 1998 (five species), *Pseudognaptorina* Kaszab, 1977 (four species), *Sintagona* Medvedev, 1998 (one species), *Tagonoides* Fairmaire, 1886 (eight species), and *Viettagona* Medvedev & Merkl, 2003 (one species) (Medvedev and Merkl 2002; Medvedev 2004; Ren et al. 2016; Li et al. 2018, 2019; Chigray 2019; Iwan and Löbl 2020; Bai et al. 2020, 2023; Ji et al. 2024). With 39 described species, *Gnaptorina* is the second most species-rich genus in the subtribe Gnaptorinina. The genus *Gnaptorina* is currently subdivided into three subgenera: *Gnaptorina* Reitter, 1887, *Austroptorina* Bai, Li & Ren, 2020, and *Hesperoptorina* Medvedev, 2009 (Medvedev 2009; Li et al. 2021). To date, immature stages of six species in three genera are described within Gnaptorinina: *Gnaptorina* Reitter, 1887 (larvae of three species), *Agnaptoria* Reitter, 1887 (larvae of two species) and *Itagonia* Reitter, 1887 (larva of one species) (Yu et al. 1996, 1999; Medvedev 2006; Zhu and Ren 2014; Ji et al. 2024). Larval morphology is important for understanding the systematics of different groups, and it has been used to support the close relationships of genera (Grebennikov and Scholtz 2004; Lawrence et al. 2011; Kamiński et al. 2019), including for supraspecific classification of the tribe Blaptini (Skopin 1960; Chigray and Kirejtshuk 2023). However, the larvae were described for only a few species of Blaptini.

In this study, we constructed a molecular phylogenetic tree for the genus *Gnaptorina* and a molecular species delimitation, combining them to verify the taxonomic status of larval specimens. These larvae belong to the five known species of the subgenus Hesperoptorina of the genus *Gnaptorina*. Larvae of these five species are described and illustrated. The present results will enrich the existing mitochondrial gene library of the tribe Blaptini and lay the foundation for future evolutionary study of the endemic insects on the Qinghai-Xizang Plateau.

## ﻿Materials and methods

### ﻿Morphological examination

In total, 170 larval samples of five species were examined for this study, which are deposited at the Museum of Hebei University, Baoding, China (**MHBU**). The larvae used for the description above were inferred to be in their older instar stage based on previous research on the larval biology of the Blaptini.

The photos were taken with the following imaging system: (a) Canon EOS 5D Mark III (Canon Inc., Tokyo, Japan) connected to a Laowa FF 100 mm F2.8 CA-Dreamer Macro 2× or Laowa FF 25 mm F2.8 Ultra Macro 2.5–10× (Anhui Changgeng Optics Technology Co., Hefei, China). (b) A Leica M205A stereomicroscope equipped with a Leica DFC450 camera (Leica Microsystems, Singapore) was controlled using the Leica application suite v. 4.3; (c) JVC KY-F75U (JVC Kenwood, Long Beach, CA, USA) digital camera attached to a Leica Z16 APO dissecting microscope (Leica Microsystems, Buffalo Grove, IL, USA) with an apochromatic zoom objective and motor focus drive, using a Syncroscopy Auto-Montage System (Synoptics, Cambridge, UK) and software. Multiple images were stacked to construct the final figures. Photographed specimens were illuminated with either an LED ring light attached to the end of the microscope column, with incidental light filtered to reduce glare, or by a gooseneck illuminator with bifurcating fibreoptics; image stacks were white-balance corrected using the system software (Synoptics, Cambridge, UK). Montaged images were edited using Adobe Photoshop v. 22.1.0 to form the final figure plates.

Label data are presented verbatim. A slash (/) separates text on different lines of label.

### ﻿Taxon sampling, DNA extraction, PCR amplification, and sequencing

Larval specimens were collected in the field from Xizang, China. To correlate the different stages, the molecular data were obtained from larval individuals.

DNA was extracted from pygopod tissue of the larva using the Insect DNA Isolation Kit (BIOMI-GA, Hangzhou, China) following the manufacturer’s protocols. The DNA extracted was stored at -20 °C. Fragment of mitochondrial molecular marker (cytochrome oxidase subunit I, COI) was amplified with the primers F 2183 and R 3014 (Folmer et al. 1994). The profile of the PCR amplification consisted of an initial denaturation step at 94 °C for 4 min, 35 cycles of denaturation at 94 °C for 45 s, annealing at 47 °C for 1.5 min, an extension at 72 °C for 1 min, and a final 8 min extension step at 72 °C. PCR was performed using TaKaRa Ex Taq (TaKaRa, Dalian, China). PCR products were subsequently checked by 1% agarose gel electrophoresis and sequencing was performed at General Biol Co. (Anhui, China). Finally, we obtained five sequences for five larvae. Detailed information for the new samples in this study is provided in Table 1.

**Table 1.** List of larvae examined in this study with the corresponding accession number.

Species	Sampling locality	Elevation (m)	Date of collection	Collector(s)	Accession number
*G.nigera*	Damxung County, Xizang, China (XZDX)		23.VII.2014	G. Ren et al.	PQ013187
*G.tishkovi*	Qomolangma, Tingri County, Xizang, China (XZDR)	4960	20.VII.2023	X. Bai et al.	PQ013187
*G.brucei*	Tingri County, Xizang, China (XZDR)	4820	28.VII.2014	G. Ren et al.	PQ013185
*G.kangmar*	Gyangzê County, Xizang, China (XZJZ)		6.VIII.2014	G. Ren et al.	PQ013184
*G.himalaya*	Qusum County, Xizang, China (XZSN)	4790	31.VII.2019	X. Li et al.	PQ013185

### ﻿Phylogenetic analyses

In total, we used 306 sequences from 88 individuals, including 301 published sequences (from 81 adults, one larva, one pupa) and five new sequences from larvae (Li et al. 2021; Ji et al. 2024). We used the previously published sequences of two species of Platyscelidini Lacordaire, 1859 as outgroups, which have been considered as close relatives of the tribe Blaptini (Kamiński et al. 2021).

The phylogenetic tree was constructed based on concatenated datasets of mitochondrial and nuclear DNA sequences (COI, Cytb, 16S, and 28S-D2) by Maximum Likelihood (ML). A best-fit model was tested according to the corrected Akaike’s Information Criterion (AIC) using ModelFinder (included in IQ-TREE) with the software PhyloSuite v. 1.2.2 (Zhang et al. 2020). The ML tree search was performed in IQ-TREE v. 1.6.8 (Nguyen et al. 2015) that was also plugged into PhyloSuite. The ML tree was inferred using an edge-linked partition model for 5000 ultrafast bootstraps (1000 replicates) (Minh et al. 2013). Support for each node is represented by ultrafast bootstrap values (uBV).

### ﻿Molecular species delimitation analyses

Recent studies have shown that some molecular species definition methods may underestimate or overestimate the number of species (Dellicour and Flot 2018; Luo et al. 2018). Hence, it has been advocated to use them in a complementary way to better assess species boundaries. Here, we used a combination of three distinct methods (ASAP, GMYC, and PTP) to assess the boundaries of species within *Gnaptorina*.

We relied on the Assemble Species by Automatic Partitioning (ASAP) approach as implemented on the online web application (https://bioinfo.mnhn.fr/abi/public/asap/asapweb.html, Puillandre et al. 2021). ASAP analysis was carried out based on COI gene fragment, and outgroups were excluded. In addition to the distance-based ASAP method, we also performed tree-based analyses using two distinct methods: the General Mixed Yule Coalescent (GMYC) model and Poisson-tree-processes (PTP) (Pons et al. 2006; Zhang et al. 2013). Accordingly, GMYC analysis was conducted on an ultrametric tree from the BEAST analysis, with all outgroups removed. The analysis was conducted in R using the package GMYC with default settings (100 trees randomly selected, 250 million generations with a burn-in of 25 million and a thinning parameter of 100). PTP analysis relied on the best-score ML tree from the IQ-TREE analysis and was carried out on the web server of the Exelixis Lab (http://species.h-its.org/ptp/) using default settings.

## ﻿Results

### ﻿Phylogenetic relationships and species delimitation

**Figure 1.**
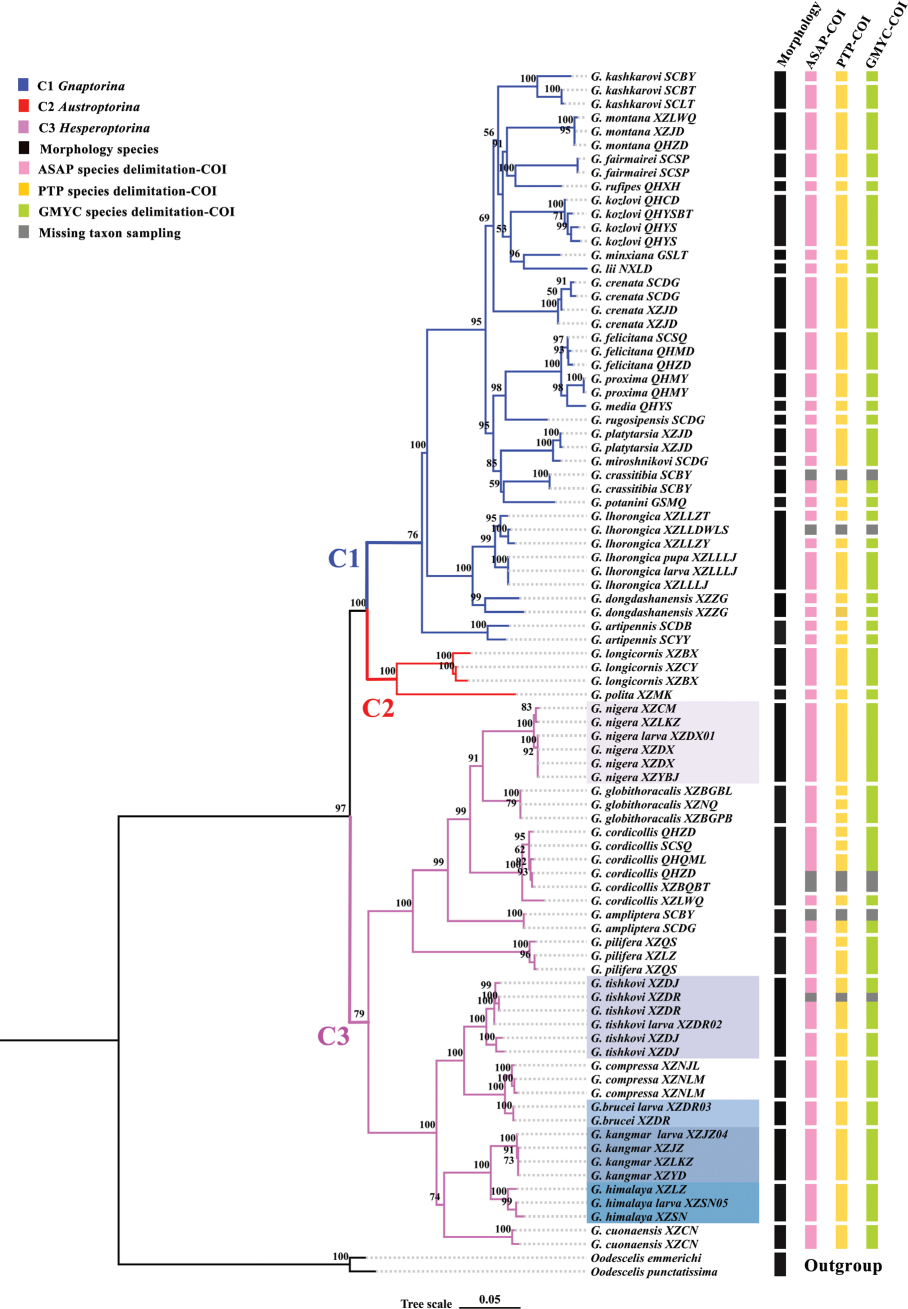
Maximum-likelihood phylogenetic tree based on 2219 bp of mitochondrial and nuclear DNA sequences (COI, Cytb, 16S, and 28S-D2) within the genus *Gnaptorina*. Vertical coloured bars delineate extant morphospecies (black), and the results of three separate molecular analyses delimiting species (pink, yellow. and pale green). For the analysis using COI gene, we used grey colour to delineate specimens for which sequencing failed.

The final, the IQ-TREE analysis yielded a topology based on concatenated dataset (2219 bp), including 306 sequences from 88 individuals (Fig. 1). The individuals of *Gnaptorina* were grouped into three well-supported clades: C1 (*Gnaptorina*, uBV = 76), C2 (*Austroptorina*, uBV = 100), and C3 (*Hesperoptorina*, uBV = 79). The monophyly of each subgenus was supported overall.

The ML tree and three molecular species delimitation methods associate the larvae and adults of different species with consistent results. Larva and known species cluster into a single well-supported clade respectively (uBV = 100). Three molecular species delimitation results showed that the samples XZDX01, XZDR02, XZDR03, XZJZ04, and XZSN05 consistently merged individuals from known species. Therefore, we conclude that the above assumption is correct: the sample XZDX01 is the larva of *G.nigera*, the sample XZDR02 is the larva of *G.tishkovi*, the sample XZDR03 is the larva of *G.brucei*, the sample XZJZ04 is the larva of *G.kangmar*, and the sample XZSN05 is the larva of *G.himalaya*.

### ﻿Key to larvae of four genera of the tribe Blaptini

**Table:** 

1	Epipharynx with six mastoids above the basal spines	**2**
–	Epipharynx with eight mastoids above the basal spines	***Itagonia***
2	Urogomphi conspicuous	**3**
–	Urogomphi inconspicuous	***Gnaptorina***
3	Labrum with not less than 6 setae on apical part	***Blaps***
–	Labrum with less than 6 setae on apical part	***Prosodes***

### ﻿Larval diagnosis of the genus *Gnaptorina* Reitter, 1887

The last segment is conical in shape; urogomphi are inconspicuous and the apex is truncated; body is brownish yellow, shiny, with ossified body wall and midline is distinct (Yu et al. 1999). Pairs of setae grow on each tergum. Labrum is transverse; apical part with six setae; anterior margin with two discal setae and lateral margin with sparse setae. Clypeus is trapezoidal, with two pairs of setae at apex and margin, respectively. Epicranial stem is Y- or V-shaped. Mandible membranous elevation with two setae. Maxillary palpi are three-segmented, with a seta inside and outside anterior margin of II. Labial palpi are two-segmented, prementum with two setae apically, mentum and submentum with sparse setae. Antennae are three-segmented, the apex of II is dilated and baseball-like, sensation circle C-shaped at apex of II, III is cylindrical, much narrower and shorter than I and II. Prothoracic leg is noticeably stronger, longer, and thicker than meso- and metathoracic legs; each tarsus has differentiated into a highly ossified tarsungulus and a weakly ossified base, with one seta internally at base and one short thick spine laterally; inner margin of each segment with a row of pectinate setae.

### ﻿Key to the species of known larvae of the genus *Gnaptorina*

**Table:** 

1	Mentum trapezoidal (Fig. 2A)	**2**
–	Mentum elongate hexagonal (Fig. 2B)	**3**
2	Median line obvious on pro- and mesonotum	***G.kangmar***
–	Median line obvious on thorax dorsally and abdominal tergite I	***G.himalaya***
3	Ocelli evident (Fig. 2C)	**4**
–	Ocelli inconspicuous (Fig. 2D)	**7**
4	Antennal segment I longer than II	**5**
–	Antennal segment I shorter than II	***G.cylindricollis***
5	Submentum with 10 setae	***G.felicitana***
–	Submentum with 6 setae	**6**
6	Median line obvious on thorax dorsally	***G.nigera***
–	Median line obvious on thorax dorsally and abdominal tergite I	***G.tishkovi***
7	Mentum with 2 setae	***G.lhorongica***
–	Mentum with 12 setae	***G.brucei***

**Figure 2.**
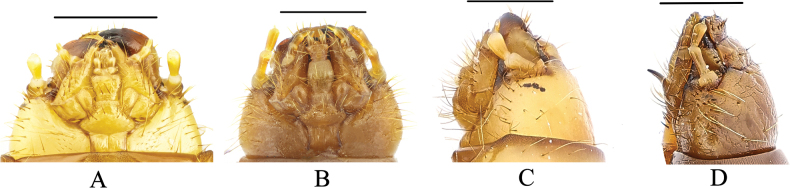
**A** head of *Gnaptorinahimalaya*, ventral view **B** head of *G.brucei*, ventral view **C** head of *G.himalaya*, lateral view **D** head of *G.brucei*, lateral view. Scale bars: 1 mm.

### ﻿Descriptions of larvae

#### ﻿ Gnaptorina nigera

Taxon classificationAnimaliaColeopteraTenebrionidae

Shi, Ren & Merkl, 2007

C8AE19FD-4E61-5B89-B37D-7DCCE2F4D760

##### Examined materials.

**Larvae.** 3 exx. (MHBU): Damxung County, Xizang/ 30°16.05’ N, 90°42.60’ E/ Alt. 4480 m/ 2023-VII-9/ Xiu-Min Li, Tong-Yang Guo leg.; 20 exx. (MHBU): Damxung County, Xizang/ 30°20.78’ N, 91°03.58’ E/ Alt. 4150m/ 2019-VIII-8/ Xiu-Min Li leg.; 31 exx. (MHBU): Yangbajain Township, Damxung County, Xizang/ 2014-VII-23/ Guo-Dong Ren, Xing-Long Bai, Jun-Sheng Shan leg.; 4 exx. (MHBU): Damxung County, Xizang/ 30°32.32’ N, 91°06.42’ E/ Alt. 4350m/ 2019-VIII-9/ Xiu-Min Li leg.; 11 exx. (MHBU): Chamda Township, Nagarzê County, Xizang/ 29°00.48’ N, 91°04.87’ E/ Alt. 4540m/ 2019-VIII-8/ Xiu-Min Li leg.; 11 exx. (MHBU): Comai County, Xizang/ 28°50.92′ N, 91°22.57′ E/ Alt. 4490m/ 2023-VII-8/ Xiu-Min Li leg.; 6 exx. (MHBU): Daglung Township, Nagarzê County, Xizang/ 28°39.48′ N, 90°28.10′ E/ Alt. 4615m/ 2014-VIII-6/ Guo-Dong Ren, Xing-Long Bai, Jun-Sheng Shan leg.

##### Description.

**Figure 3.**
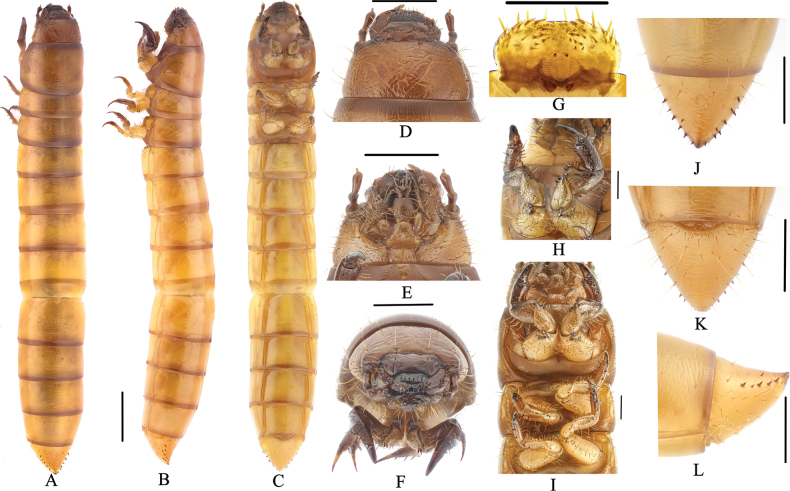
Larva of *Gnaptorinanigera* Shi, Ren & Merkl, 2007 **A** habitus, dorsal view **B** habitus, lateral view **C** habitus, ventral view **D** head, dorsal view **E** head, ventral view **F** head, vertex view **G** epipharynx **H** prothoracic leg **I** legs **J** pygopods, dorsal view **K** pygopods, ventral view **L** pygopods, lateral view. Scale bars: 2 mm (**A–C**); 1 mm (**D–F, J–L**); 0.5 mm (**G, H, I**).

***Body*** (Fig. 3A–C). Larvae length 17.2–17.5 mm, width 2.1–2.5 mm. Body subcylindrical; last segment conical; body brownish yellow, shiny, body wall ossified; median line obvious on thorax dorsally; pairs of setae grow on each tergum.

***Head*** (Fig. 3E, D–G). Prognathous, slightly narrower than width of prothorax; labrum transverse; apical part with six setae; anterior margin with two discal and six slender lateral marginal setae; epipharynx with sparse setae on lateral margin, with two basal spines on central area, between the basal spines with four mastoids, with six mastoids above the basal spines (Fig. 3G); mandible left-right unsymmetrical, membranous elevation with two setae; clypeus transverse, trapezoidal, dark brown, with two pairs of setae at apex and margin respectively (Fig. 3D, F). Epicranial stem Y or V-shaped; frons convex, dark brown throughout, with sparse long setae on lateral margins, with four pairs of setae at apex (a pair on anterior margin, a pair on middle margin, two pairs on posterior margin) (Fig. 3D, F). Ocelli evident, two parallel rows arranged transversely (Fig. 3B). Maxillary palpi (Fig. 3E) three-segmented, cylindrical, and conical at apex; I widest, II longest, I as long as III. Labial palps two-segmented, short; II conical; prementum with two setae on anterior margin, apex with two long setae, lateral sides with four or five long setae; mentum convex, hexagonal, base of mentum straight; mentum more slender than prementum, lateral margins with four or five long setae, posterior margin with four setae; submentum with six setae on mid-posterior part (three on left, three on right). Antennae three-segmented, cylindrical at apex; I longest, as wide as II; III shortest and narrowest (Fig. 3B, D–F).

***Thorax*** (Fig. 3A). Thoracic segments parallel-sided, with transverse plicae. Pronotum and mesonotum with two pairs of elongate setae on anterior and posterior margin. Metanotum with two pairs of setae on anterior margin and a pair of setae on posterior margin. Anterior and posterior border of pronotum with brown longitudinal stripes, with pair of irregular brown spots on tergum, posterior border of mesonotum and metanotum with a brown longitudinal stripe. Pronotum longest, 2.40 × as long as mesonotum, 1.70 × as long as metanotum, mesonotum shortest.

***Legs*** (Fig. 3H, I). Protarsungulus strongly sclerotised, sharp, claw-like; protarsungulus with a strong, long seta on inner side and a strong, short spine on outer side at base. Profemora and protibiae gradually narrowing towards apex; inner margin setal formula of prothoracic leg 4(3): 6(3): 2(2); outer margin of tibiae with two setae; outer margin of femora with two setae; trochanter with two setae (Fig. 3G). Mesotarsus with a strong, long seta on inner side and a strong, short spine on outer side at base; inner margin setal formula of mesothoracic leg 3–4(2–3): 4(2): 2(2); outer margin of tibiae with two spines; outer margin of femora with two spines; outer margin of trochanters with two spines. Metatarsus with two short, broad spines at base, inner margin setal formula of metathoracic leg 3(2): 4(2): 2(2), outer margin of tibiae with two short spines, outer margin of femora with two spines, outer margin of trochanters with two spines. Meso- and metathoracic legs shorter than prothoracic one, meso- and metathoracic legs tarsungulus highly ossified, hooked, with dense setae (Fig. 3I).

***Abdomen*** (Fig. 3C, J–L). Approximately 3.10 × as long as thorax; abdominal segments I–VIII subcylindrical, with transverses plicae; ventral side of abdominal segment I with six pairs of setae on each side (five pairs of setae near anterior and a pair of setae near posterior) and 14 setae on anterior margins, ventral side of abdominal segments II–VIII with three pairs of setae on anterior, middle, and posterior margin of lateral margins, respectively (Fig. 3C). Last segment conical, 0.91 × as long as VIII, distinctly narrower than VIII; surface of convex disc with sparse long setae in ventral view, with a row of short spines on each side (six spines on left, seven spines on right); last segment dorsally flattened; urogomphi inconspicuous and apex truncated, with two short spines (Fig. 3J–L).

***Spiracles*** (Fig. 3C). Lateral margins of abdominal segments I–VIII and mesothorax each with a pair of spiracles, mesothoracic spiracles much larger than abdominal one, lateral margins of abdominal segments I–VIII with almost equal-sized spiracles, rounded.

#### ﻿ Gnaptorina tishkovi

Taxon classificationAnimaliaColeopteraTenebrionidae

Medvedev, 1998

5F2F7427-0208-5807-BA30-5F669FC41790

##### Examined materials.

**Larvae.** 14 exx. (MHBU): Qomolangma, Tingri County, Xizang/ 28°11.33′ N, 86°49.80′ E/ Alt. 4960m/ 2023-VII-20/ Xing-Long Bai, Quan-Yu Ji, Jian Song leg.; 5 exx. (MHBU): Tingri County, Xizang/ 28°36.68′ N, 87°07.78′ E/ Alt. 4270m/ 2014-VII-24/ Guo-Dong Ren, Xing-Long Bai, Jun-Sheng Shan leg.; 5 exx. (MHBU): Tingri County, Xizang/ 28°27.58′ N, 87°37.15′ E/ Alt. 4480m/ 2019-VIII-16/ Xiu-Min Li leg.; 10 exx. (MHBU): Dinggyê County, Xizang/ 28°08.48′ N, 87°42.45′ E/ Alt. 4700m/ 2014-VIII-4/ Guo-Dong Ren, Xing-long Bai, Jun-Sheng Shan leg.; 5 exx. (MHBU): Dinggyê County, Xizang/ 2014-VIII-4/ Guo-Dong Ren leg.

##### Description.

**Figure 4.**
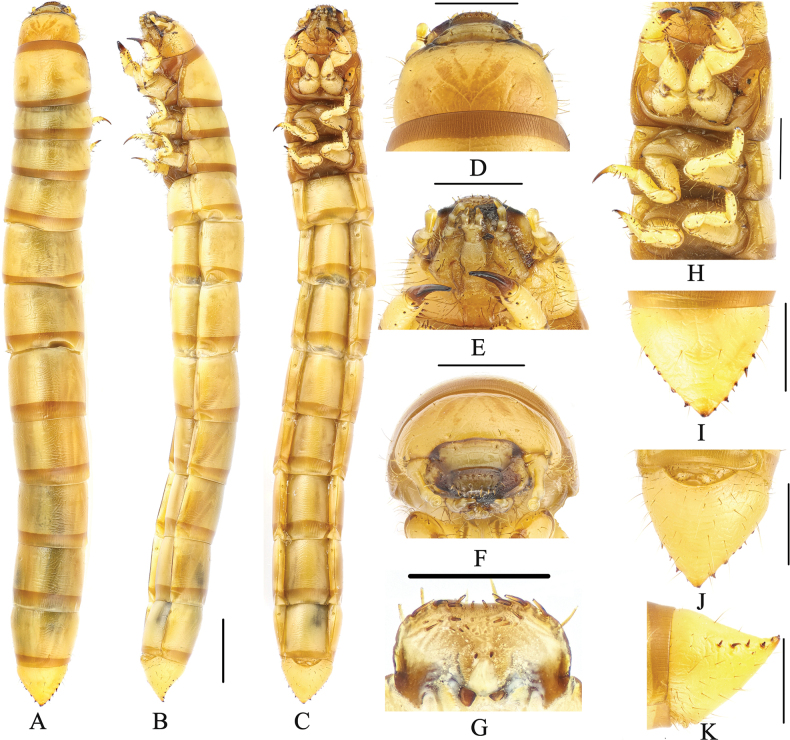
Larva of *Gnaptorinatishkovi* Medvedev, 1998 **A** habitus, dorsal view **B** habitus, lateral view **C** habitus, ventral view **D** head, dorsal view **E** head, ventral view **F** head, vertex view **G** epipharynx **H** legs **I** pygopods, dorsal view **J** pygopods, ventral view **K** pygopods, lateral view. Scale bars: 2 mm (**A–C**); 1 mm (**D–F, H–K**); 0.5 mm (**G**).

***Body*** (Fig. 4A–C). Larvae length 21.8–23.5 mm, width 2.4–3.0 mm. Body yellowish brown, shiny, body wall ossified; median line obvious on on thorax dorsally and abdominal tergite I.

***Head*** (Fig. 4B, D–G). Labrum transverse; apical part with six setae; anterior margin with two discal and six slender lateral marginal setae; epipharynx with sparse setae on lateral margin, with two basal spines on central area, between the basal spines with four mastoids, with six mastoids above the basal spines (Fig. 4G); mandible left-right unsymmetrical, membranous elevation with two setae (Fig. 4D, F). Epicranial stem Y-shaped, epicranial stem with a pair of pale brown patterns on distal margin; frons convex, with sparse long setae on lateral margins, with four pairs of setae at apex (a pair on upper margin, a pair on middle margin, two pairs on mid-posterior margin) (Fig. 4D, F). Posterior margin of middle part of frontal pale brown covered. Ocelli evident (Fig. 4B). Maxillary palpi (Fig. 4E) three-segmented, cylindrical, and conical at apex; I widest, II longest. Labial palps two-segmented, short; II conical; prementum with two setae on anterior margin, apex with two long setae, lateral sides with two long setae; mentum convex, hexagonal; mentum more slender than prementum, posterior margin with four long setae; submentum with six setae on posterior margin (three on left, three on right). Antennae three-segmented, cylindrical at apex; I longest and widest; III shortest and narrowest (Fig. 4B, D–G).

***Thorax*** (Fig. 4A). Pronotum and metanotum with two pairs of elongate setae on anterior margin and a pair of setae on posterior margin. Mesonotum with a pair of setae on anterior, middle, and posterior margin. Anterior and posterior borders of pronotum with brown longitudinal stripes, posterior border of mesonotum and metanotum with a brown longitudinal stripe. Pronotum longest, 1.96 × as long as mesonotum, 1.61 × as long as metanotum, mesonotum shortest.

***Legs*** (Fig. 4H). Protarsungulus with a strong, long seta on inner side and a strong, short spine on outer side at base. Profemora and protibiae gradually narrowing towards apex; inner margin setal formula of prothoracic leg 3–4(1–2):6(3):2(2); outer margin of tibiae with two strong, short spines; outer margin of femora with two setae; trochanter with two short setae. Mesotarsus with a strong, long seta on inner side and a strong, short spine on outer side at base; inner margin setal formula of mesothoracic leg 4:5(1):2(2); outer margin of tibiae with two spines; outer margin of femora with two spines; outer margin of trochanters with two spines and one seta. Metatarsus with a strong, long seta on inner side and a strong, short spine on outer side at base; inner margin setal formula of metathoracic leg 3–4(2):4(3):2(2), outer margin of tibiae with two spines, outer margin of femora with two spines, outer margin of trochanters with two spines and one seta.

***Abdomen*** (Fig. 4A, C). Not constricted between VIII and IX segments. Approximately 4.20 × as long as thorax; abdominal segments I–VIII subcylindrical, with transverses plicae; ventral side of abdominal segment I with sparse setae on anterior and lateral margins, with four setae on posterior margin (two on left, two on right); ventral side of abdominal segments II–VIII with three pairs of setae on anterior, middle, and posterior margin of lateral margins, respectively. Last segment conical, 0.87 × as long as VIII, distinctly narrower than VIII; surface of convex disc with sparse long setae in ventral view, with a row of short spines on each side (six spines each on left and right); last segment dorsally flattened; urogomphi inconspicuous and apex truncated, with two short spines (Fig. 4I–K).

***Spiracles*** (Fig. 4C). Mesothoracic spiracles are almost twice size of abdominal segment I spiracles; lateral margins of abdominal segments I–VIII and mesothorax each with a pair of oval spiracles, abdominal segment I spiracles largest, abdominal segments I–VIII spiracles gradually shrinking.

#### ﻿ Gnaptorina brucei

Taxon classificationAnimaliaColeopteraTenebrionidae

Blair, 1923

9252CF90-660B-53E3-9C68-DD9829CE3986

##### Examined materials.

**Larvae.** 2 exx. (MHBU): Rongxar Township, Tingri County, Xizang/ 28°10.92′ N, 86°29.25′ E/ Alt. 4820m/ 2014-VII-28/Guo-Dong Ren, Xing-Long Bai, Jun-Sheng Shan leg.

##### Description.

**Figure 5.**
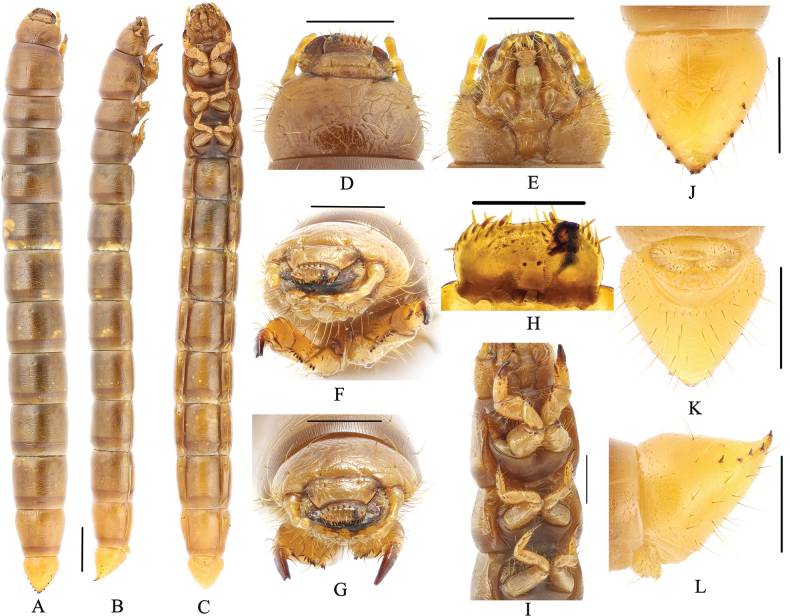
Larva of *Gnaptorinabrucei* Blair, 1923 **A** habitus, dorsal view **B** habitus, lateral view **C** habitus, ventral view **D** head, dorsal view **E** head, ventral view **F, G** head, vertex view **H** epipharynx **I** legs **J** pygopods, dorsal view **K** pygopods, ventral view **L** pygopods, lateral view. Scale bars: 2 mm (**A–C**); 1 mm (**D–G, I–L**); 0.5 mm (**H**).

***Body*** (Fig. 5A–C). Larvae length 24.5–25.5 mm, width 2.2–2.5 mm, comparatively thin. Body yellowish brown, shiny, body wall ossified; median line obvious on thorax dorsally and abdominal tergite I.

***Head*** (Fig. 5B, D–H). Labrum transverse; apical part with six setae; anterior margin with two discal and six slender lateral marginal setae; epipharynx with sparse setae on lateral margin, with two basal spines on central area, between the basal spines with four mastoids, with six mastoids above the basal spines (Fig. 5H); mandible left-right unsymmetrical, membranous elevation with two setae (Fig. 5D, E). Epicranial stem Y-shaped; frons convex, frons with densely long setae on lateral margins, with four pairs of setae at apex (a pair on anterior margin, a pair on mid-anterior margin, two pairs on mid-posterior margin) (Fig. 5D). Ocelli inconspicuous (Fig. 5B). Maxillary palpi (Fig. 5E) three-segmented, cylindrical, and conical at apex; I widest, II longest. Labial palps two-segmented, short; II conical; prementum with two setae on anterior margin, apex with two long setae, lateral sides with four long setae; mentum convex, hexagonal; mentum more slender than prementum, lateral margin with five or six long setae, posterior margin with one long setae; submentum with eight setae on posterior margin. Antennae three-segmented, cylindrical at apex; I longest and widest; III shortest and narrowest.

***Thorax*** (Fig. 5A). Each thoracic tergum with two pairs of elongate setae on anterior and posterior margin. Anterior and posterior borders of pronotum with brown longitudinal stripes; posterior border of mesonotum and metanotum with a brown longitudinal stripe. Pronotum longest, 1.70 × as long as mesonotum, 1.45 × as long as metanotum, mesonotum shortest.

***Legs*** (Fig. 5I). Protarsungulus with a strong, long seta on inner side and a strong, short spine on outer side at base. Profemora and protibiae gradually narrowing towards apex; inner margin setal formula of prothoracic leg 5(3): 6–7(4): 2(2); outer margin of tibiae with one short seta and one strong, short spine; outer margin of femora with two setae; trochanter with three setae. Mesotarsus with a strong, long seta on inner side and a strong, short spine on outer side at base; inner margin setal formula of mesothoracic leg 3(3): 5(3): 2(2); outer margin of tibiae with two spines; outer margin of femora with two spines; outer margin of trochanters with one spine and two setae. Metatarsus with a strong, long seta on inner side and a strong, short spine on outer side at base; inner margin setal formula of metathoracic leg 3(2): 5–6(3): 2(2), outer margin of tibiae with two spines, outer margin of femora with two spines, outer margin of trochanters with one spine and two setae.

***Abdomen*** (Fig. 5A, C). Constricted between VIII and IX segments. Approximately 3.54 × as long as thorax; abdominal segments I–VIII subcylindrical, with transverses plicae; ventral side of abdominal segment I with sparse setae on anterior and lateral margins, with two setae on posterior margin; ventral side of abdominal segments II–VIII with two pairs of setae on lateral margin. Last segment conical, 0.79 × as long as VIII, distinctly narrower than VIII; last segment surface of convex disc with sparse long setae in ventral view, with a row of short spines on each side (four spines on left, six spines on right); urogomphi inconspicuous and upturned slightly, with two short spines (Fig. 5J–L).

***Spiracles*** (Fig. 5C). Lateral margins of abdominal segments I–VIII and mesothorax each with a pair of oval spiracles, mesothoracic spiracles largest, abdominal segments I–VIII spiracles gradually shrinking.

#### ﻿ Gnaptorina himalaya

Taxon classificationAnimaliaColeopteraTenebrionidae

Shi, Ren & Merkl, 2007

ECA4EE4F-9401-5F45-B517-73235E0AF2FD

##### Examined materials.

**Larvae.** 2 exx. (MHBU): Garyü Countyside, Qusum County, Xizang/ 28°50.25’ N, 91°59.90’ E/ Alt. 4790m/ 2019-VII-31/ Xiu-Min Li leg.; 4 exx. (MHBU): Zag La, Comai County, Xizang/ 2019-VII-31/ Guo-Dong Ren leg.

##### Description.

**Figure 6.**
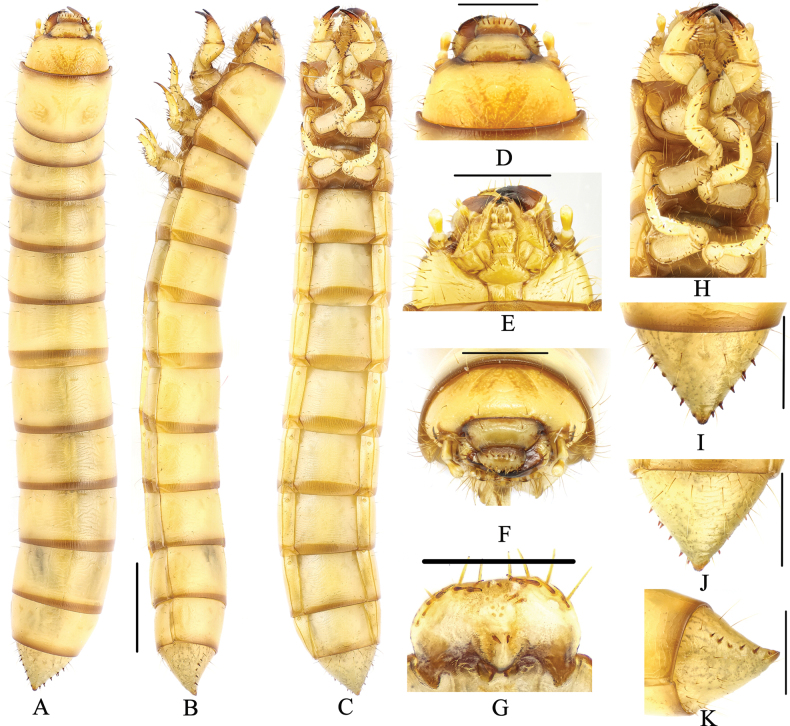
Larva of *Gnaptorinahimalaya* Shi, Ren & Merkl, 2007 **A** habitus, dorsal view **B** habitus, lateral view **C** habitus, ventral view **D** head, dorsal view **E** head, ventral view **F** head, vertex view **G** epipharynx **H** legs **I** pygopods, dorsal view **J** pygopods, ventral view **K** pygopods, lateral view. Scale bars: 2 mm (**A–C**); 1 mm (**D–F, H–K**); 0.5 mm (**G**).

***Body*** (Fig. 6A–C). Larvae length 16.8–22.0 mm, width 2.1–2.3 mm, thick. Body yellowish brown, shiny, body wall ossified; median line obvious on thorax dorsally and abdominal tergite I.

***Head*** (Fig. 6B, D–G). Labrum transverse; apical part with six setae; anterior margin with two discal and six slender lateral marginal setae; epipharynx with sparse setae on lateral margin, with two basal spines on central area, between the basal spines with four mastoids, with three pairs of mastoids above the basal spines; mandible left-right unsymmetrical, membranous elevation with two setae (Fig. 6D, F, G). Epicranial stem Y or V-shaped; frons convex, with sparse setae on lateral margins, with four pairs of setae at apex (a pair on anterior margin, a pair on middle margin, two pairs on posterior margin) (Fig. 6D, F). Ocelli evident, three parallel rows arranged transversely (Fig. 6B). Maxillary palpi (Fig. 6E) three-segmented, cylindrical, and conical at apex; I widest, II longest. Labial palps two-segmented, short; II conical; prementum shorter than mentum, with two setae on anterior margin, apex with two long setae; mentum convex, trapezoidal, base of mentum straight; mentum wide and short, posterior margin with 4–6 long setae; submentum with nine setae on middle margin. Antennae three-segmented, cylindrical at apex; I nearly as long as II; III shortest and narrowest (Fig. 6B, D–G).

***Thorax*** (Fig. 6A). Pronotum with four pairs of setae (two pairs of setae on anterior margin, a pair of setae on middle margin, a pair of setae on posterior margin); mesonotum with three pairs of long setae, a pair on anterior margin, two pairs on middle; metanotum with four pairs of setae, two pairs on anterior margin, two pairs on middle. Anterior and posterior borders of pronotum with brown longitudinal stripes, and a pair of pale brown irregular spots; posterior border of mesonotum and metanotum with a brown longitudinal stripe. Pronotum longest, 2.80 × as long as mesonotum, 2.06 × as long as metanotum, mesonotum shortest.

***Legs*** (Fig. 6H). Protarsungulus with a strong, long seta on inner side and a strong, short spine on outer side at base. Profemora and protibiae gradually narrowing towards apex; inner margin setal formula of prothoracic leg 5(4): 6(2–3): 2(2); outer margin of tibiae with one strong seta and one short spine; outer margin of femora with two setae; trochanter with three setae. Mesotarsus with a strong, long seta on inner side and a strong, short spine on outer side at base; inner margin setal formula of mesothoracic leg 2–4(2–3):5(2–3):2(2); outer margin of tibiae with two spines; outer margin of femora with two spines; outer margin of trochanters with one spine and two setae. Metatarsus with two strong, short spines at base; inner margin setal formula of metathoracic leg 3(2–3): 4(2):2(2), outer margin of tibiae with two spines, outer margin of femora with two spines, outer margin of trochanters with one spine and two setae.

***Abdomen*** (Fig. 6A, C). Not constricted between VIII and IX segments. Approximately 3.91 × as long as thorax; abdominal segments I–VIII subcylindrical, with transverses plicae; ventral side of abdominal segment I with 11 setae on anterior margin and 5–7 on each side, with two pairs of setae on posterior margin; ventral side of abdominal segment II with six pairs of setae (four pairs of setae on lateral margin, two pairs of setae on posterior margin); ventral side of abdominal segments III–VIII with four pairs of setae on lateral margin (two pairs of setae on mid-lateral margin, two pairs of setae on posterior margin). Last segment conical, 0.87 × as long as VIII, distinctly narrower than VIII; last segment surface of convex disc with sparse long setae in ventral view, with a row of short spines each side (five spines on left, five spines on right); last segment dorsally flattened, urogomphi inconspicuous, with one short spines (Fig. 6I–K).

***Spiracles*** (Fig. 6C). Lateral margins of abdominal segments I–VIII and mesothorax each with a pair of oval spiracles, mesothorax spiracles much larger than abdominal spiracles, abdominal segments I–VIII spiracles gradually shrinking.

#### ﻿ Gnaptorina kangmar

Taxon classificationAnimaliaColeopteraTenebrionidae

Shi, Ren & Merkl, 2007

BD497C50-1170-5A59-89D3-C389B2E9E952

##### Examined materials.

**Larvae.** 27 exx. (MHBU): Nai Chin Kangsang Snow Mountain, Xizang/ 28°53.90’ N, 90°09.85’ E/ Alt. 5030m/ 2014-VIII-6/ Guo-Dong Ren, Xing-Long Bai, Jun-Sheng Shan leg.; 21 exx. (MHBU): Gyangzê County, Xizang/ 2014-VIII-6/ Guo-Dong Ren, Xing-Long Bai, Jun-Sheng Shan leg.

##### Description.

**Figure 7.**
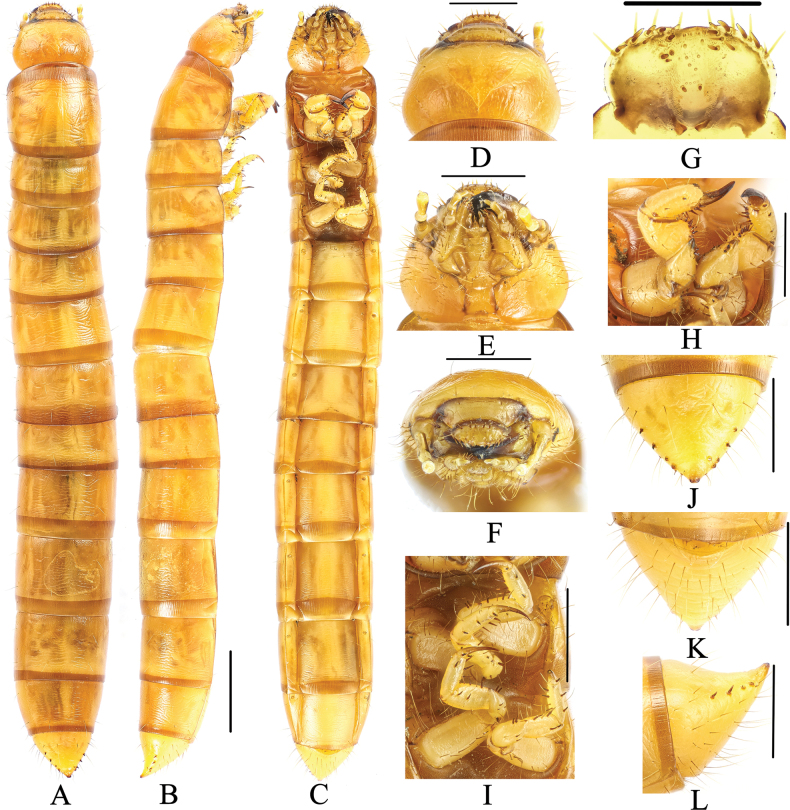
Larva of *Gnaptorinakangmar* Shi, Ren & Merkl, 2007 **A** habitus, dorsal view **B** habitus, lateral view **C** habitus, ventral view **D** head, dorsal view **E** head, ventral view **F** head, vertex view **G** epipharynx **H** prothoracic leg, lateral view **I** mesothoracic and metathoracic legs, lateral view **J** pygopods, dorsal view **K** pygopods, ventral view **L** pygopods, lateral view. Scale bars: 2 mm (**A–C**); 1 mm (**D–F, H–L**); 0.5 mm (**G**).

***Body*** (Fig. 7A–C). Larvae length 19.2–20.0 mm, width 2.3–2.5 mm, moderately thickened. Body yellowish brown, shiny, body wall ossified; median line obvious on pronotum and mesonotum.

***Head*** (Fig. 7B, D–G). Labrum transverse; apical part with six setae; anterior margin with two discal and six slender lateral marginal setae; epipharynx with sparse setae on lateral margin, with two basal spines on central area, between the basal spines with four mastoids, with six mastoids above the basal spines; mandible left-right unsymmetrical, membranous elevation with two setae (Fig. 7D, E, G). Epicranial stem Y-shaped; frons convex, with sparse setae on lateral margins, with four pairs of setae at apex (a pair on anterior margin, a pair on middle margin, two pairs on mid-posterior margin) (Fig. 7D, F). Ocelli evident, two parallel rows arranged transversely (Fig. 7B). Maxillary palpi (Fig. 7E) three-segmented, cylindrical, and conical at apex; I widest, II longest. Labial palps two-segmented, short; II conical; prementum short, with two setae on anterior margin, apex with two long setae; mentum convex, trapezoidal, base of mentum straight; mentum wide and short, lateral margin with five or six long setae, mid-posterior margin with two long setae; submentum with nine setae on middle margin. Antennae three-segmented, cylindrical at apex; I longest; III shortest and narrowest (Fig. 7B, D–G).

***Thorax*** (Fig. 7A). Pronotum and mesonotum with four pairs of setae (two pairs of setae on anterior margin, two pairs of setae on posterior margin); metanotum with three pairs of long setae, a pair on mid-anterior margin, two pairs on middle. Anterior and posterior borders of pronotum with brown longitudinal stripes; posterior border of mesonotum and metanotum with a brown longitudinal stripe. Pronotum longest, 2.03 × as long as mesonotum, 1.89 × as long as metanotum, mesonotum shortest.

***Legs*** (Fig. 7H, I). Protarsungulus with a strong, long seta on inner side and a strong, short spine on outer side at base. Profemora and protibiae gradually narrowing towards apex; inner margin setal formula of prothoracic leg 5(1): 7(5–6): 2(2); outer margin of tibiae with two strong and short spines; outer margin of femora with two setae; trochanter with three setae. Mesotarsus with a strong, long seta on inner side and a strong, short spine on outer side at base; inner margin setal formula of mesothoracic leg 4(2): 4(2): 2(2); outer margin of tibiae with two spines; outer margin of femora with two spines; outer margin of trochanters with two spines and one seta (Fig. 7H). Metatarsus with a strong, long seta on inner side and a strong, short spine on outer side at base; inner margin setal formula of metathoracic leg 4(3): 5(2): 2(2), outer margin of tibiae with two spines, outer margin of femora with two spines, outer margin of trochanters with two spines and one seta.

***Abdomen*** (Fig. 7A, C). Not constricted between VIII and IX segments Approximately 2.68 × as long as thorax; abdominal segments I–VIII subcylindrical, with transverses plicae; ventral side of abdominal segment I with 12 setae on anterior margin and six setae on each side, with two pairs of setae on posterior margin; ventral side of abdominal segment II with seven pairs of setae (five pairs of setae on lateral margin, two pairs of setae on posterior margin); ventral side of abdominal segments III–VIII with three pairs of setae (two pairs of setae on mid-lateral margin, a pairs of setae on posterior margin); ventral side of abdominal segment VIII with four pairs of setae on lateral margin and two pairs of setae on posterior margin. Last segment conical, 0.91 × as long as VIII, distinctly narrower than VIII; last segment surface of convex disc with sparse long setae in ventral view, with a row of short spines on each side (five spines on left, five spines on right); last segment dorsally flattened, urogomphi inconspicuous, with two short spines (Fig. 7J–L).

***Spiracles* (Fig. 7C**). Lateral margins of abdominal segments I–VIII and mesothorax each with a pair of oval spiracles, mesothorax spiracles much larger than abdominal spiracles, abdominal segments I–VIII spiracles gradually shrinking.

## ﻿Discussion

Molecular species identification has become an important approach in insect taxonomy (Tautz et al. 2002; Hebert et al. 2003; Meier et al. 2006; Rodriguez et al. 2022). These approaches are capable of establishing correlations between larval and adult stages through DNA sequences, and of providing valuable reference information for larval taxonomy (Kamiński et al. 2019). Most *Gnaptorina* species are distributed in the high-elevation areas of the Qinghai-Xizang Plateau, where they usually have restricted areas of distribution (Bai et al. 2020; Li et al. 2021; Ji et al. 2024). Therefore, it is usually hard to obtain larvae and pupae through laboratory rearing because of the difficulty in replicating the natural conditions of *Gnaptorina* in the wild. In this study, the larval samples were directly collected from the field, whose classification is challenging due to the lack of larval information on the known species. Our results clearly provided a tool to help associate the larva with known or unknown adults, which successfully resolved the problem of larval taxonomic status. In addition, the results of molecular species delimitation are consistent with previous studies (Li et al. 2021). However, molecular species delimitation was performed based on 88 samples of 32 species (82% of the known species). We did not have a high number of specimens per species on average, which could lead to an increase in the number of inferred MOTUs. Yet molecular species delimitation was performed only based on COI gene fragments per species in this study, requiring a cautious approach to any taxonomic changes. For these taxa, we identified distinctive morphological characters that could support their status as separate species. The molecular phylogenetic results revealed that the larval specimens all belong to the subgenus Hesperoptorina. Before the present study, the larval information in only known for three species of the subgenus Gnaptorina (*Gnaptorinacylindricollis* Reitter, 1889, *Gnaptorinafelicitana* Reitter, 1887, and *Gnaptorinalhorongica* Li, 2024) were recorded. The immature stages of more genera and species need to be properly documented in order to develop an applicable system of the larval and pupal taxonomy in the tribe Blaptini.

## Supplementary Material

XML Treatment for Gnaptorina nigera

XML Treatment for Gnaptorina tishkovi

XML Treatment for Gnaptorina brucei

XML Treatment for Gnaptorina himalaya

XML Treatment for Gnaptorina kangmar

## ﻿Additional information

### Author ORCIDs

Bao-Yue Ji hhttps://orcid.org/0000-0002-9250-5481

Tong-Yang Guo https://orcid.org/0009-0004-1880-7045

Mei-Chang Gu https://orcid.org/0009-0003-6617-4572

Guo-Dong Ren https://orcid.org/0000-0001-5808-9122

Xiu-Min Li https://orcid.org/0000-0003-0575-1869

### Data availability

All of the data that support the findings of this study are available in the main text.
